# Supplementary material for: Machine learning predicts treatment response to nusinersen in non-sitter spinal muscular atrophy (SMA)
Source: J Neuromuscul Dis. 2026 Oct 20:22143602261496327. Online ahead of print. doi: 10.1177/22143602261496327 (PMC13683824; doi:10.1177/22143602261496327)
Supplement: Supplementary file 1 [file sj-pdf-1-jnd-10.1177_22143602261496327.pdf]

HINE Kick:

For inclusion in the model, ability to kick on the HINE was defined as a score of 1/2/3/4 vs 0. Starting in supine, the child scores on this item if they are able to demonstrate kicking (i.e. any movement of the hip or knee). The minimal movement for scoring at least a 1 is horizontal gravity eliminated movement (i.e. leg sliding while resting on the bed).

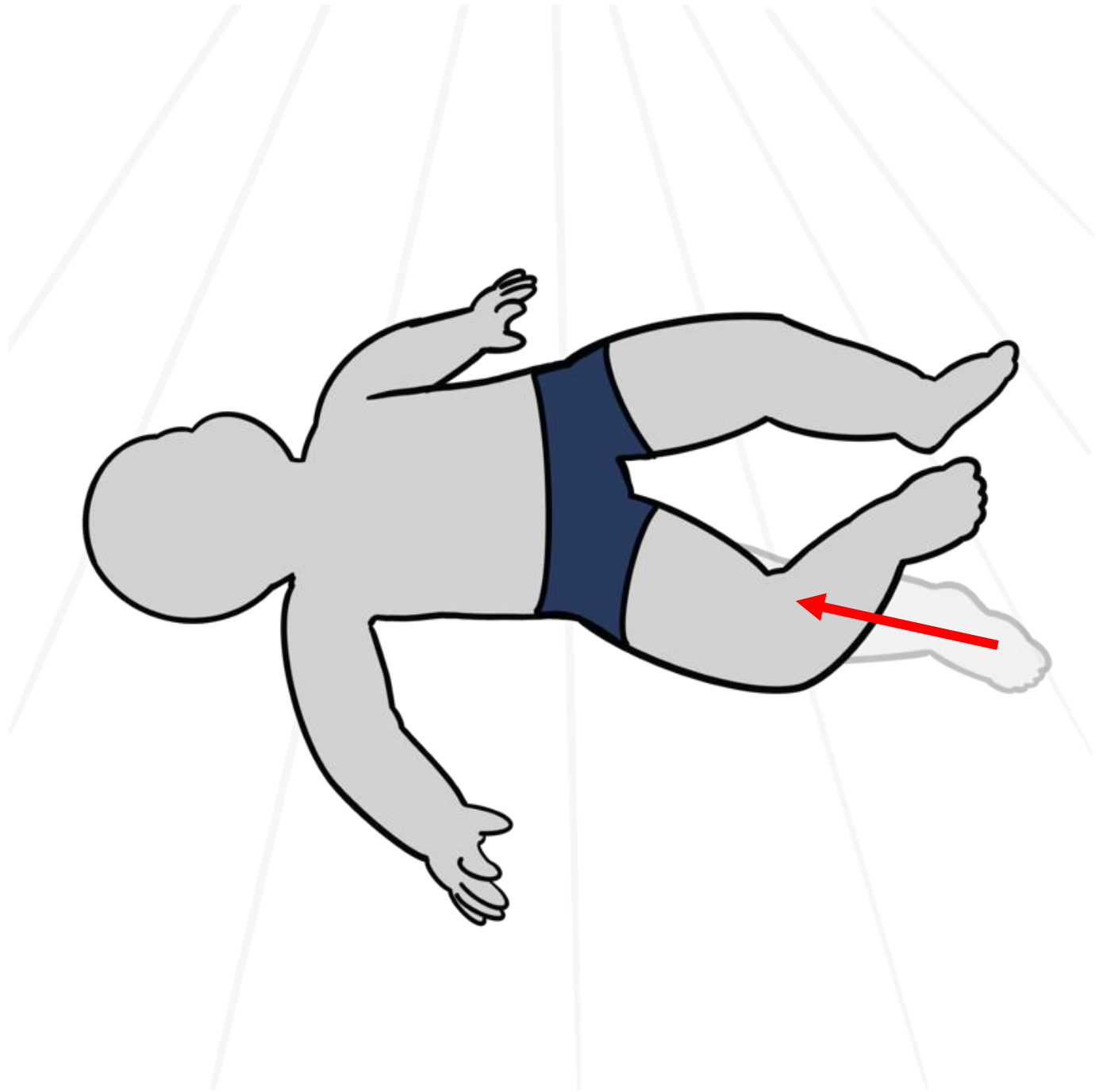

Figure 1

CHOP item 1: Spontaneous movement (upper extremity)

For inclusion in the model, a score of 4 is required on this item. Starting in supine, the child should be observed to have antigravity shoulder movement where the elbows lift off the surface). This is shown in Figure 2. Movements where the hand and arm lift off the surface, but the elbow does not (as shown in Figure 3) are insufficient.

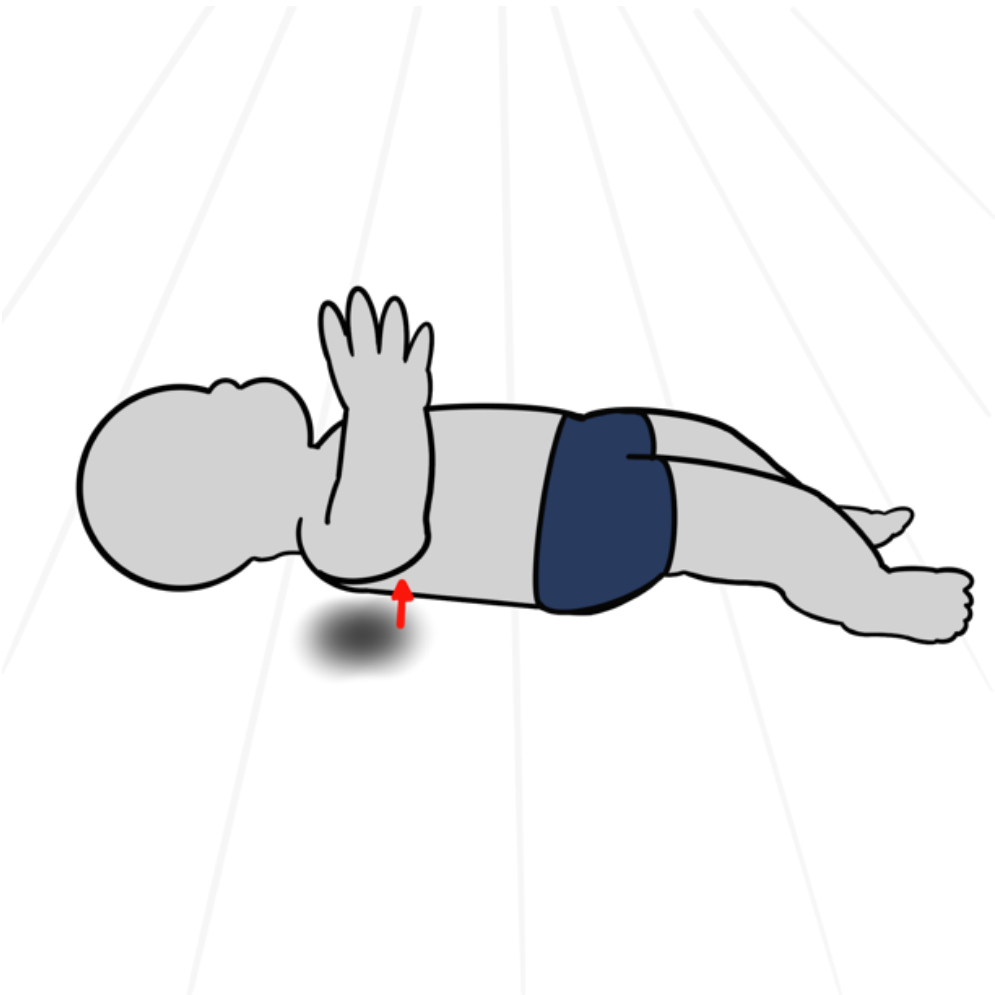

Figure 2

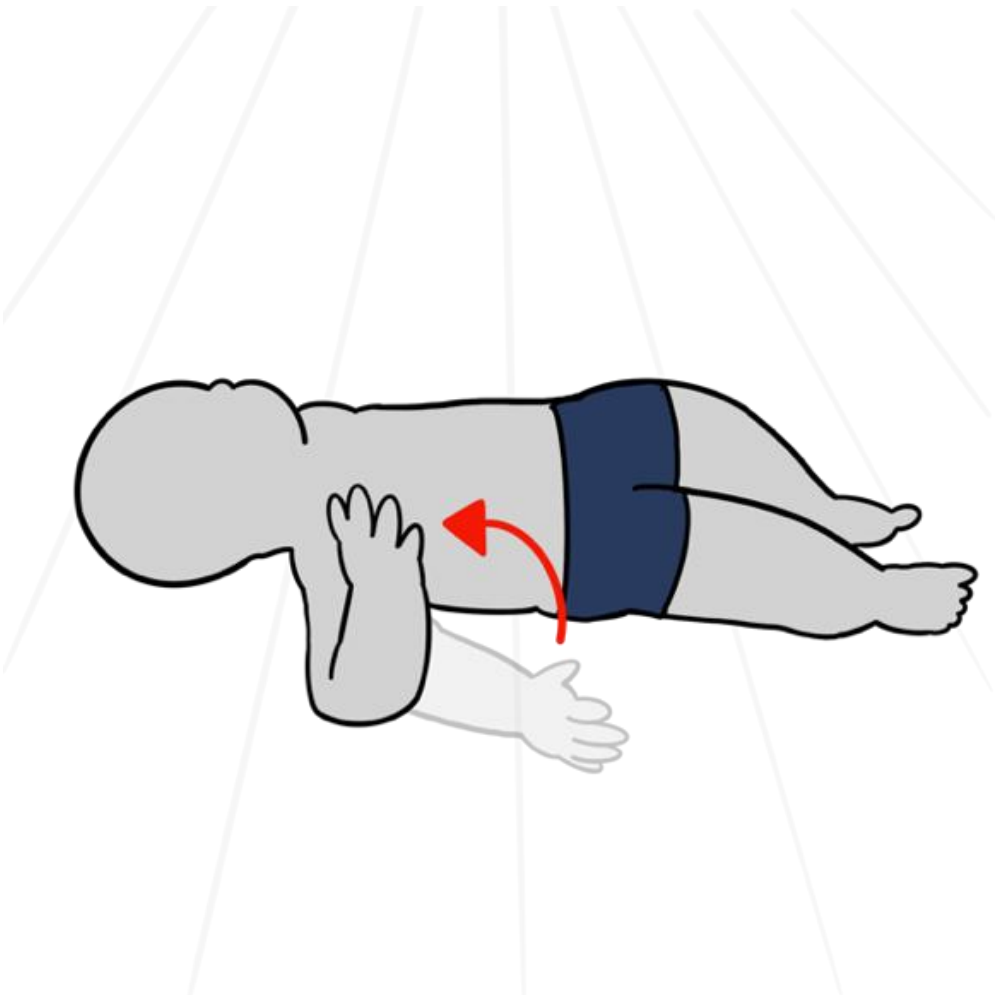

Figure 3

CHOP item 2: Spontaneous movement (lower extremity)

For inclusion in the model, a score of 2, 3, or 4 is required on this item. Starting in supine, the child should be observed to have any knee or hip movements, whether that is gravity eliminated knee or hip movement (score of 2), antigravity hip rotation (score of 3- as shown in Figure 4) or antigravity hip movement where the foot clears off the mat (score of 4- as shown in Figure 5).

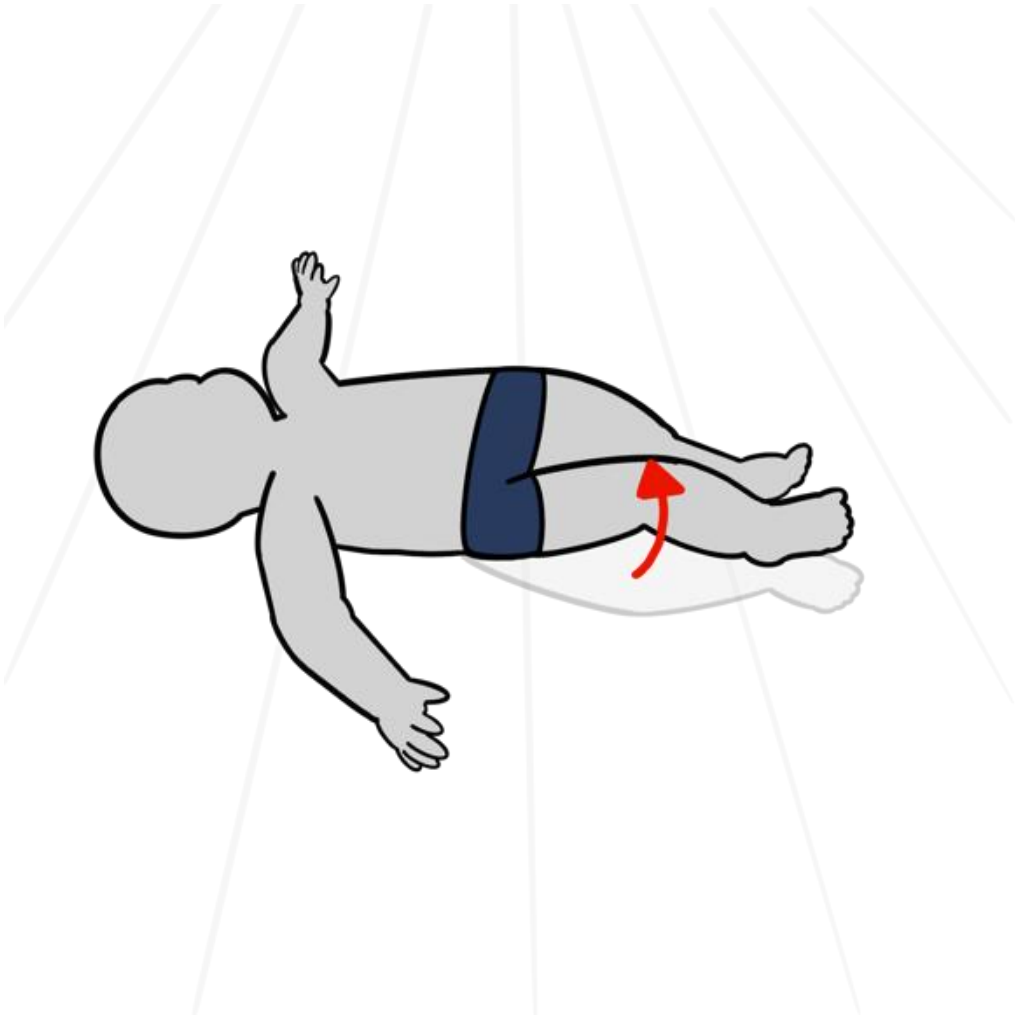

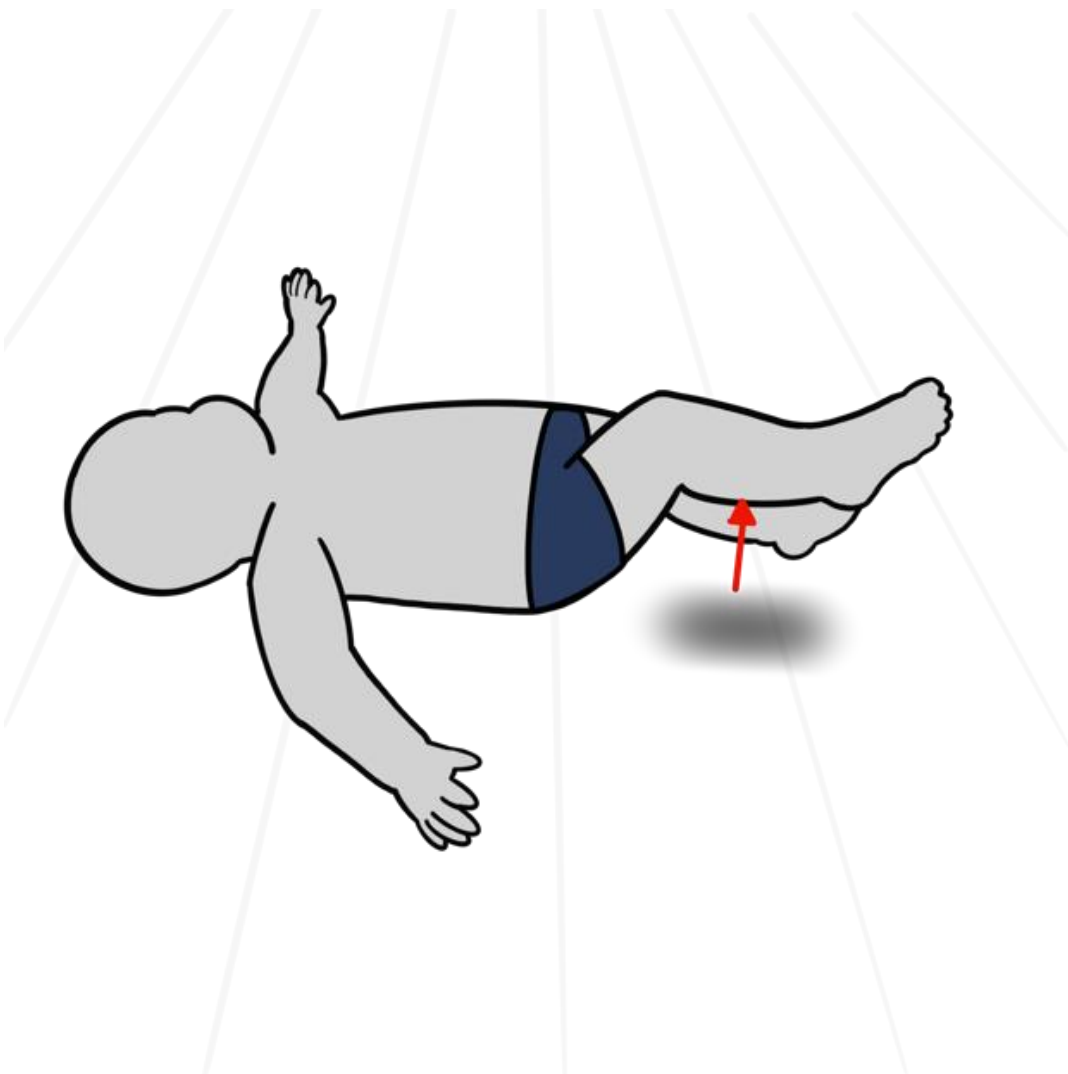

Figure 4

Figure 5

CHOP 3: Hand grip

For inclusion in the model, a score of 4 is required on this item. Starting in supine with the patient’s arm at their side, the patient must grasp the assessor’s finger, and maintain grasp as their arm is raised until the shoulder is raised off the bed, as shown in Figure 6. It is insufficient for the grip to be maintained when only the elbow is lifted, as shown in Figure 7.

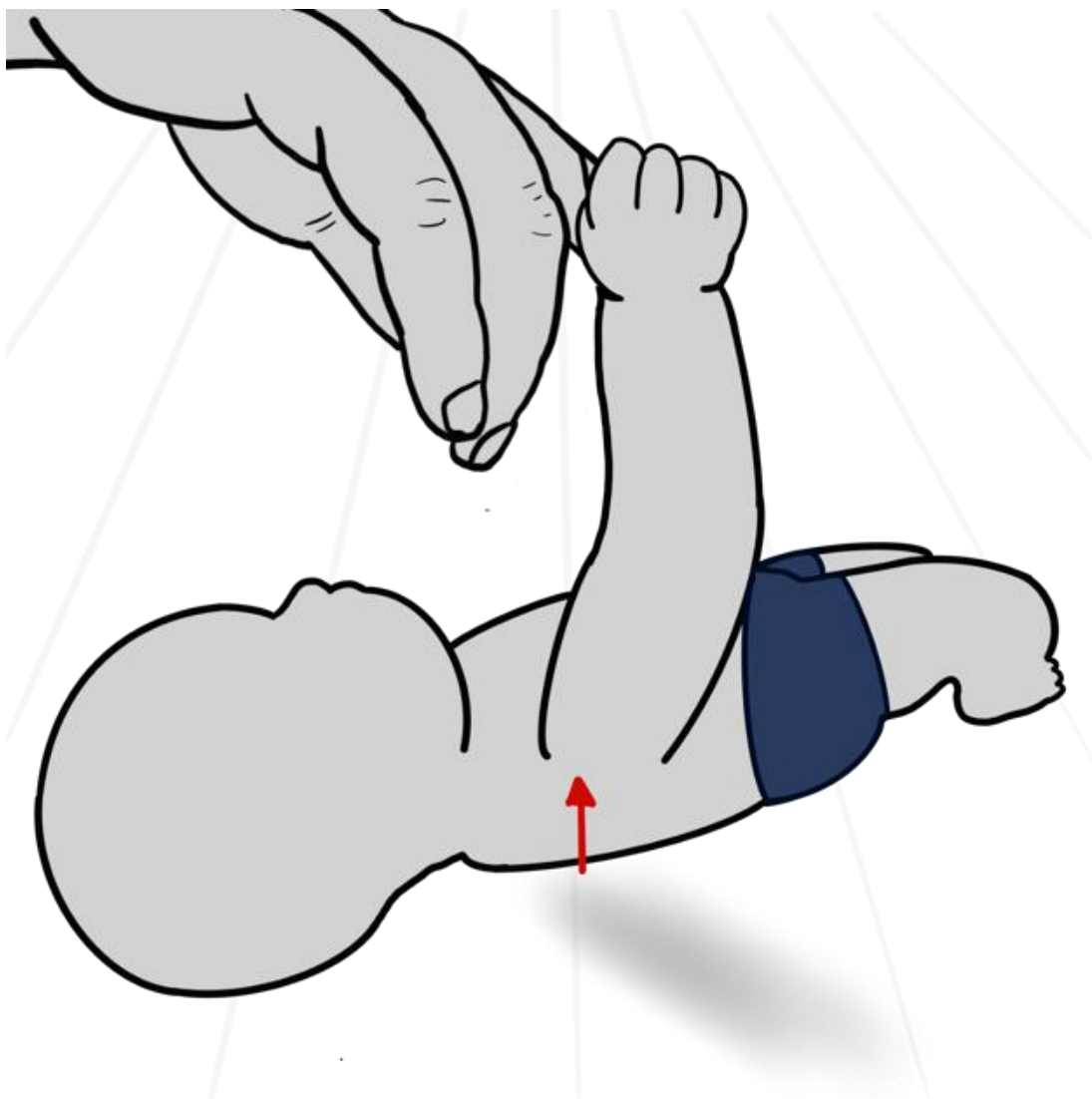

Figure 6

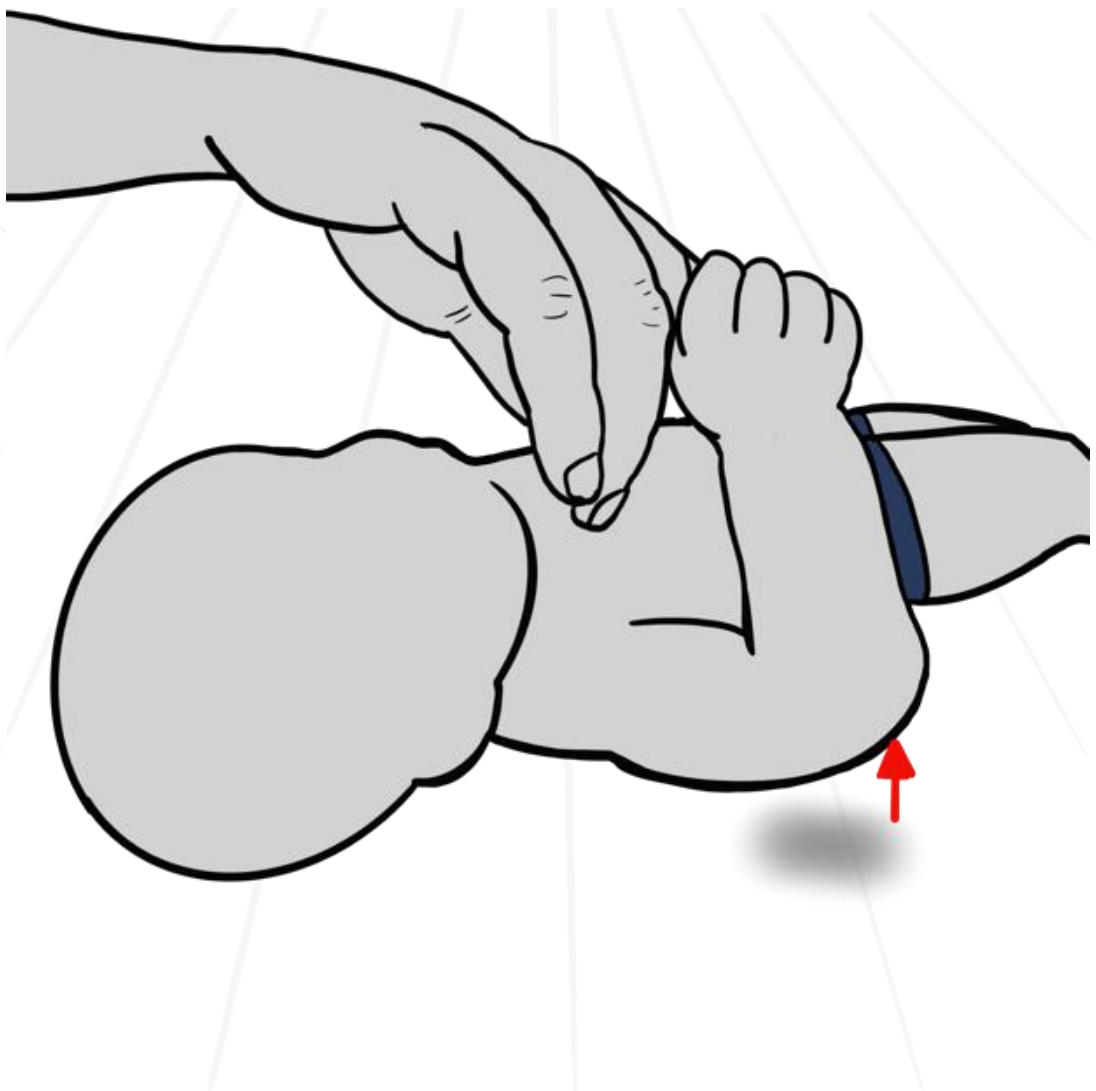

Figure 7

CHOP 7: Rolling elicited from the arms

For inclusion in the model, a score of 3 or 4 is required on this item. Starting in supine, traction is applied to the arms across the body and paused at midline. If the patient is only able to roll to side-lying (Figure 8) or rolls to prone but does not clear their arm (Figure 9), they would score a 2 which is insufficient for inclusion in the mode. If the patient is able to complete the roll to prone, with (score of 4- s in Figure 10) or without (score of 3- as in Figure 11) head righting.

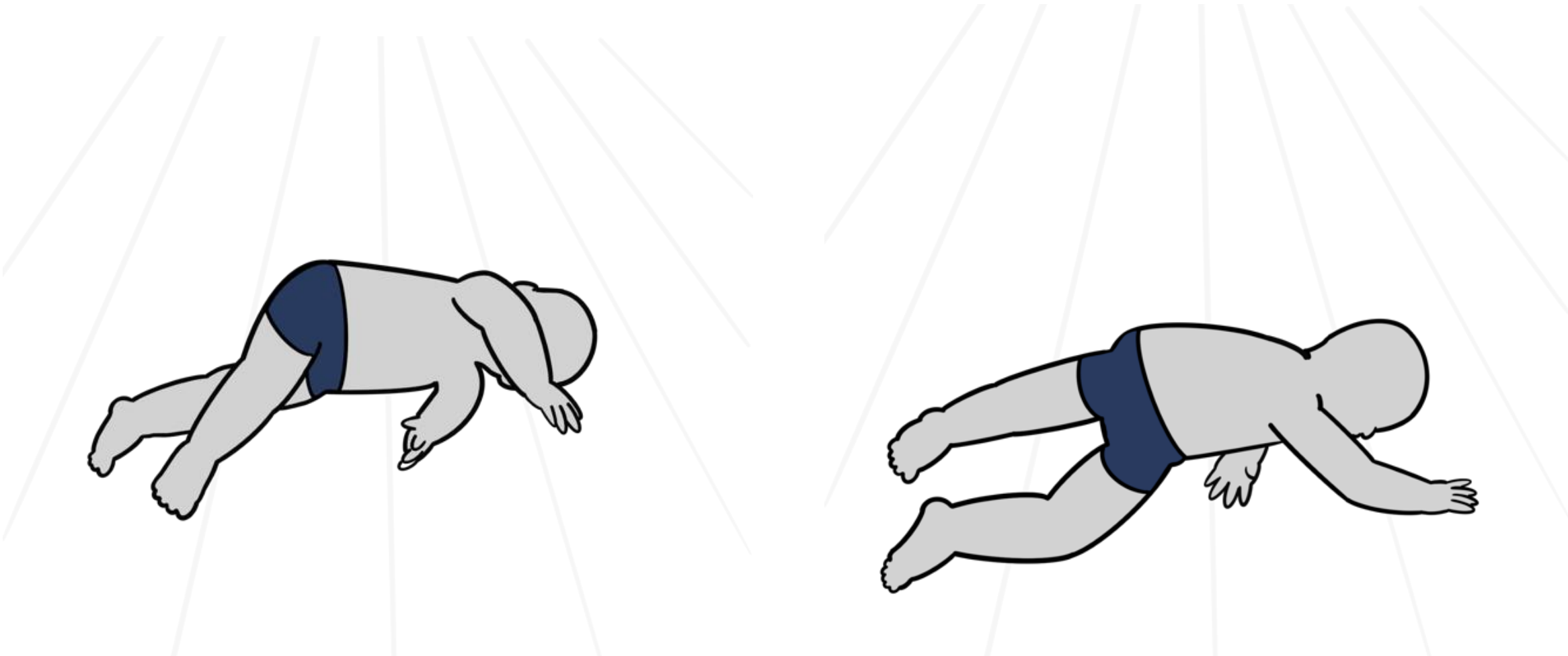

Figure 8

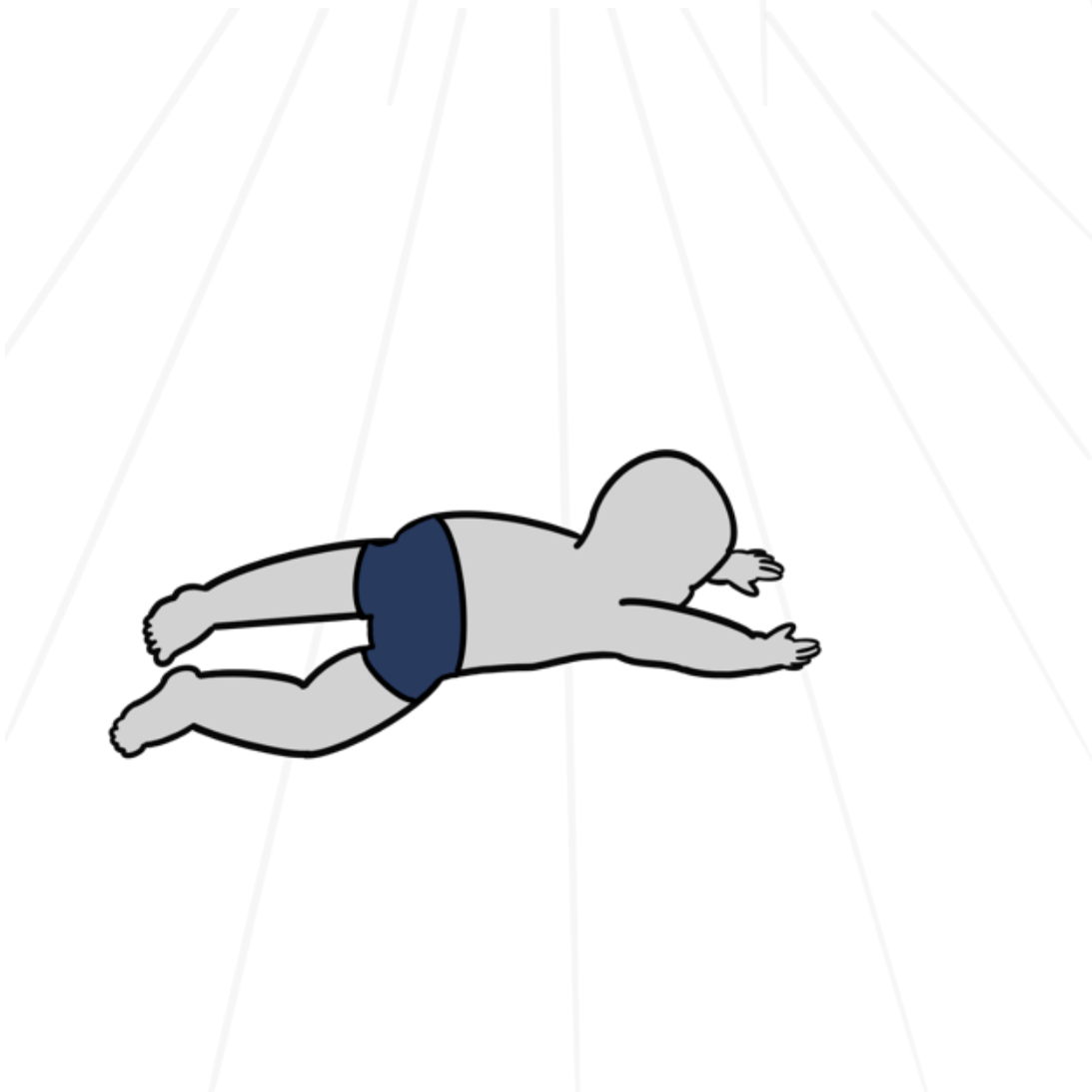

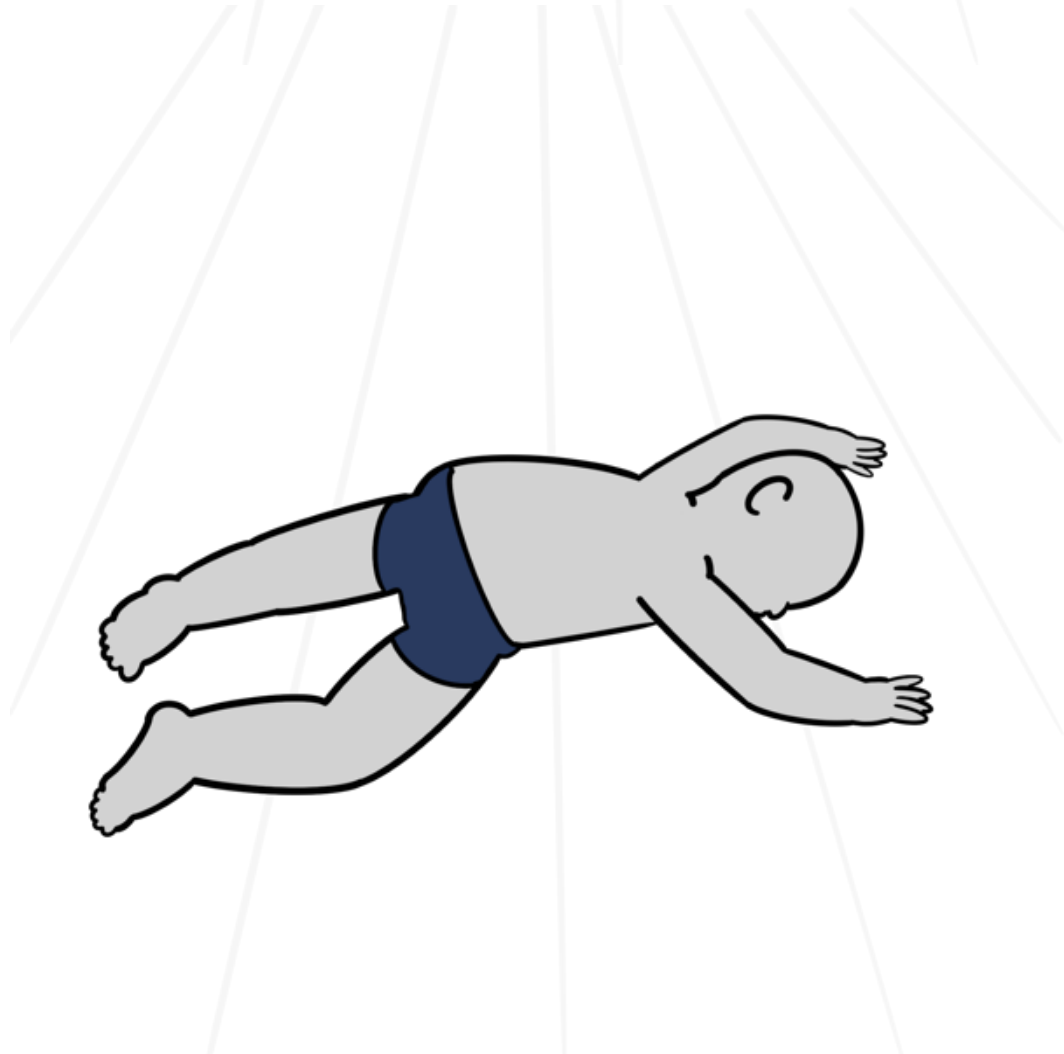

Figure 9

Figure 10

Figure 11

CHOP 8: Shoulder flexion and elbow flexion and horizontal abduction

For inclusion in the model, a score of 4 is required on this item. Starting in side-lying, the child must achieve antigravity shoulder movement, i.e. is able to hold the upper arm off the mat in front of them, as shown in Figure 12. If there was only shoulder flexion, which is gravity eliminated (i.e. the hand stays on the mat), as shown in Figure 13, then this is insufficient to score.

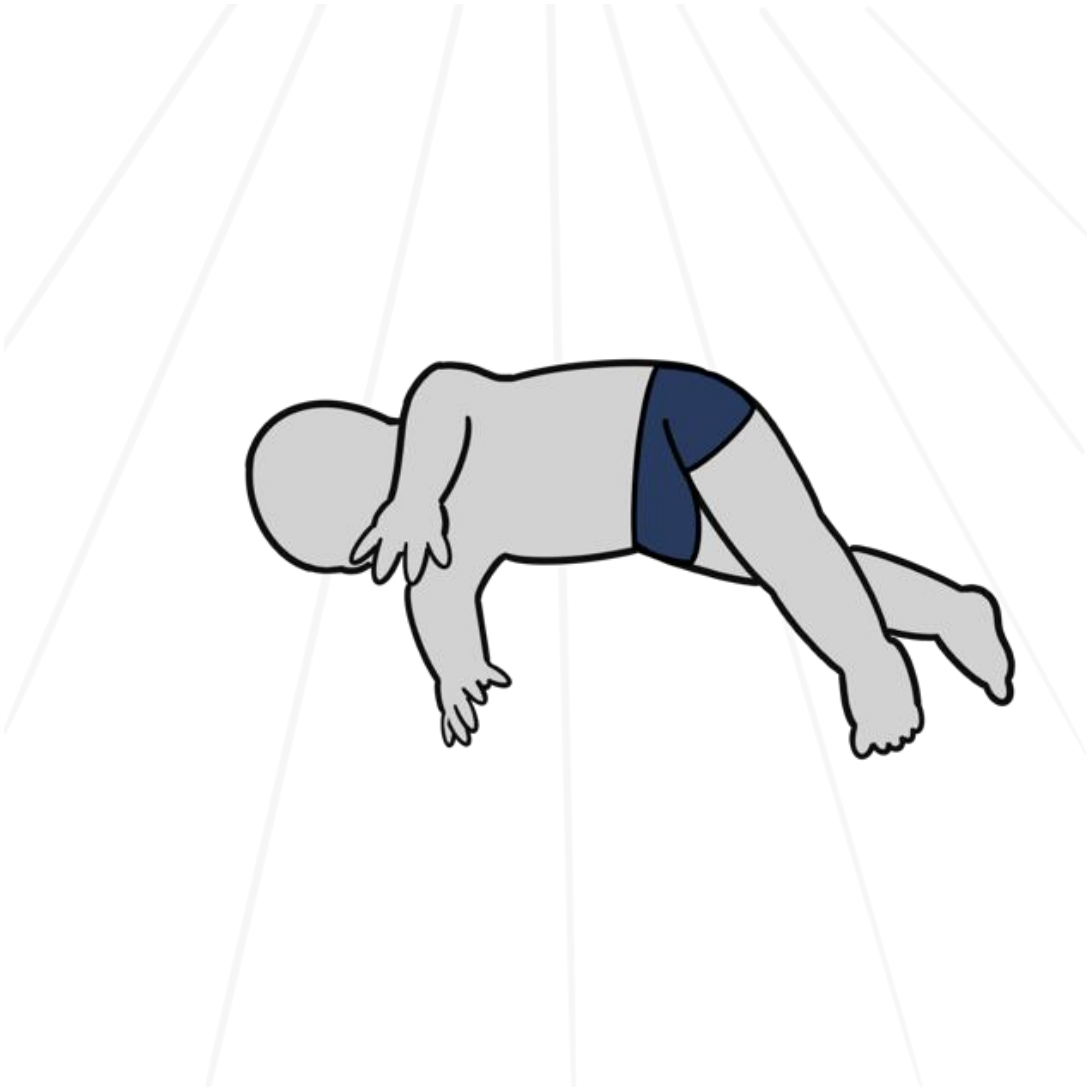

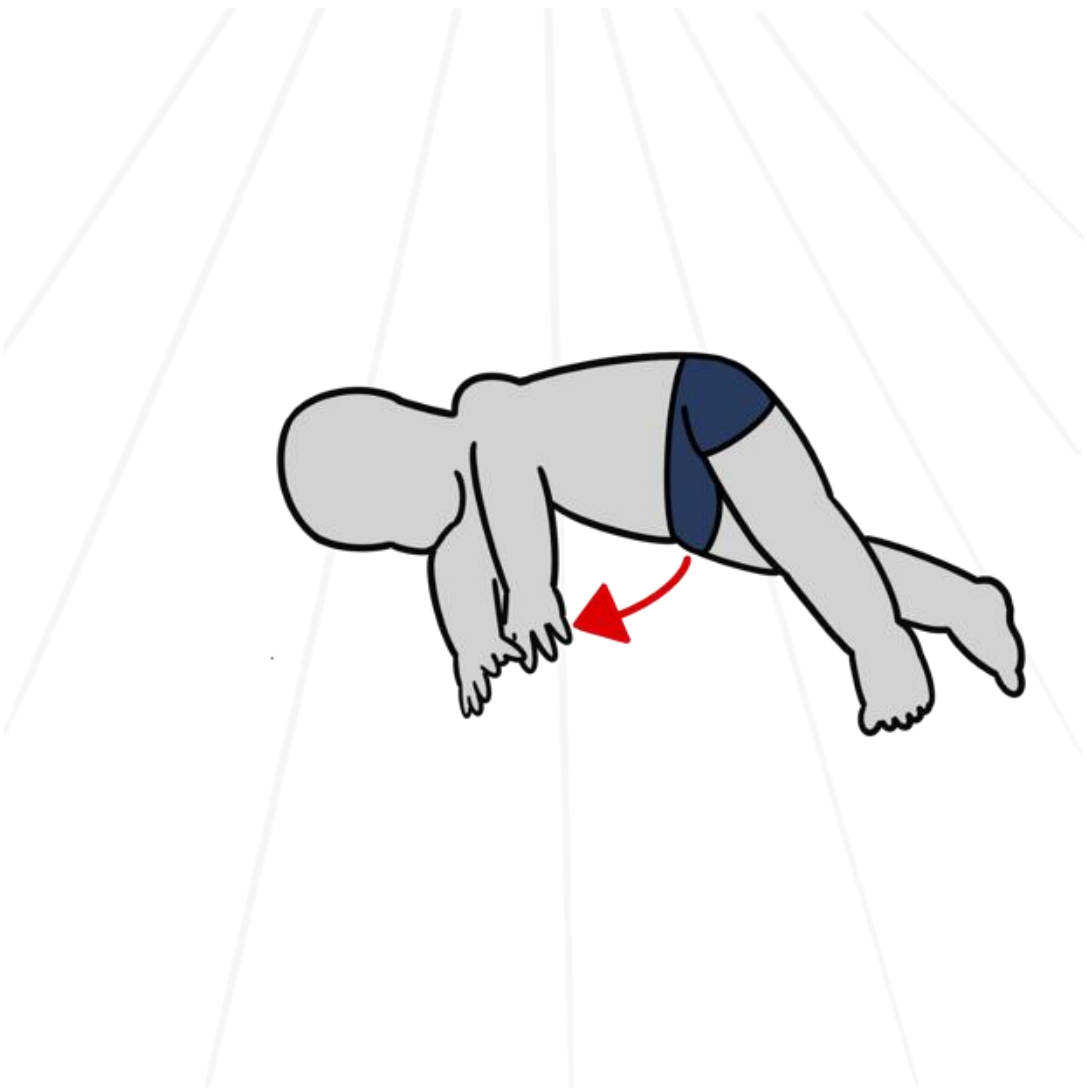

Figure 12

Figure 13
